# Performance evaluation of a commercial multiplex pathogen panel for detection of bacteria in sputum specimens from non-ICU patients with suspected lower respiratory tract infection

**DOI:** 10.1128/spectrum.02111-25

**Published:** 2025-09-15

**Authors:** Madeline Gemoules, Tristan T. Timbrook, Elizabeth Neuner, Rebekah E. Dumm, Tamara Krekel

**Affiliations:** 1 Department of Pharmacy, Barnes-Jewish Hospital 21737, St. Louis, Missouri, USA; 2 Department of Pharmacotherapy, University of Utah College of Pharmacy 15531, Salt Lake City, Utah, USA; 3 Division of Laboratory and Genomic Medicine, Department of Pathology and Immunology, Washington University School of Medicine in St. Louis 12275 , St. Louis, Missouri, USA; University of Mississippi Medical Center, Jackson, Mississippi, USA

**Keywords:** BioFire, diagnostic performance, lower respiratory tract infection, sputum, bacteria

## Abstract

Rapid diagnostic testing can improve pathogen detection and lead to targeted antibiotics. The BioFire FilmArray Pneumonia Panel (BFPP) is a multiplex PCR that has displayed strong concordance with traditional microbiologic techniques. However, most existing literature focuses on deep respiratory specimens, and there is sparse literature on performance in sputum specimens. This retrospective, single-center study included adult patients between 1 September 2022 and 31 August 2024 who had collection of a BFPP with standard of care (SOC) culture from a sputum specimen on a non-intensive care unit (ICU) floor or in the emergency department if admitted to a non-ICU floor. Out of 189 BFPPs performed on 189 sputum specimens, a total of 141 bacterial targets were detected. Between the BFPP and SOC culture, the overall positive percent agreement and negative percent agreement (NPA) were 96.3% and 54.9%, respectively. The positive predictive value (PPV) was 26.3% while the negative predictive value was 98.9%. Patients with greater than 24 h of antibiotic exposure prior to BFPP collection had a lower PPV compared to patients with less than 24 h or no exposure (13.6% vs 29.6% vs 30.4%). The lowest concordance was observed for *Haemophilus influenzae* (15.4%), *Moraxella catarrhalis* (18.2%), *Streptococcus pneumoniae* (19%), and *Staphylococcus aureus* (22.7%), several of which are fastidious in culture. BFPP showed a high NPA, with all bacterial targets having an NPA greater than 90%, except *H. influenzae* (82%). Based on these data, a negative BFPP in sputum specimens could help to rule out a bacterial pneumonia, but the benefit of a positive test remains unclear.

**IMPORTANCE:**

This study evaluates the BioFire FilmArray Pneumonia Panel (BFPP) by comparing its performance to standard of care cultures exclusively in sputum specimens from non-intensive care unit patients with suspected lower respiratory tract infection. Findings show an overall high positive percent agreement and negative predictive value but a low negative percent agreement and positive predictive value, suggesting that a negative test in sputum specimens could be beneficial when attempting to rule out a bacterial infection, but the benefit of a positive test remains unclear, particularly if common airway colonizing bacteria are detected and at low semi-quantitative thresholds. Clinical symptoms should guide test interpretation in patients with positive BFPP results but negative culture growth.

## INTRODUCTION

Lower respiratory tract infections (LRTIs) contribute to significant morbidity and mortality, being ranked globally as the fifth leading cause of death in 2021 (1). Traditional techniques for pathogen detection and susceptibility reporting can fail to detect all relevant pathogens and can take several days to result, which often leads to the initiation of empiric broad-spectrum antibiotics based on patient risk factors (2, 3). Rapid bacterial diagnostic testing has the potential to improve pathogen detection and lead to prompt targeting of antimicrobials and ultimately minimize unnecessary exposure to broad-spectrum antibiotics (4). Current community-acquired, hospital-acquired, and ventilator-associated pneumonia (CAP, HAP, and VAP, respectively) guidelines by the Infectious Diseases Society of America (IDSA) and the American Thoracic Society include minimal recommendations in relation to rapid bacterial diagnostic testing with the exception of nasal methicillin-resistant *Staphylococcus aureus* (MRSA) cultures/polymerase chain reaction (PCR) to aid in decisions surrounding anti-MRSA coverage (2, 3, 5). A 2023 CAP pathway released from the IDSA to facilitate implementation of the guidelines suggests using molecular testing for bacteria if timely pathogen determination allows for more directed therapy or discontinuation of antibiotics (6).

The BioFire FilmArray Pneumonia Panel (BFPP) is a multiplex PCR panel that can rapidly identify 33 targets, including 18 bacteria, 8 viruses, and 7 antimicrobial resistance genes. In the regulatory approval study comparing BFPP to routine standard of care (SOC) culture, the BFPP displayed an overall sensitivity for bacterial targets that ranged from 76.9% to 100% for sputum-like specimens, including 478 sputum and 447 endotracheal aspirate (ETA) specimens, and from 57.1% to 100% for bronchoalveolar lavage (BAL)-like specimens, including 821 BAL and 83 mini-BAL specimens which did not require bronchoscopy. The overall specificity for bacterial targets ranged from 89.1% to 99.3% for sputum-like specimens and from 93.1% to 99.5% for BAL-like specimens (7, 8). Since sputum-like specimens included both sputum and ETA specimens, the performance for sputum specimens alone is unknown and may be lower than when combined with higher quality ETA specimens (9, 10).

Since approval, most real-world literature surrounding performance of the BFPP includes critically ill, intensive care unit (ICU) patients and mostly includes ETA, BAL, and bronchial washing specimens (11). The limited literature on the diagnostic performance of the BFPP in non-critically ill patients and sputum specimens is described in a recent BFPP performance systematic review and meta-analysis identifying performance in only six studies with 259 total sputum specimens out of 30 studies with 8453 patients. These limited number of sputum specimens had an overall sensitivity for bacterial targets of 86% (95% confidence interval [CI] 73–94%) and an overall specificity for bacterial targets of 95% (95% CI 93–96%) (11). Given the paucity of data in sputum specimens from non-ventilated, floor-level patients with suspected LRTI, we evaluated the analytical performance of the BFPP as compared to SOC culture in this patient population.

## MATERIALS AND METHODS

### Study design and population

This retrospective, single-center study included adult patients admitted to Barnes-Jewish Hospital, a 1,400-bed urban academic medical center, between 1 September 2022 and 31 August 2024. Patients were selected who had collection of a sputum specimen with BFPP and aerobic Gram stain and culture while on a non-ICU floor or while in the emergency department if subsequently admitted to a non-ICU floor. Clinical diagnosis of LRTI by the patient care team was assumed given ordering of the BFPP and the challenges in applying the criteria for LRTI (12, 13). No restrictions for ordering the BFPP were in place during the study timeframe, and ordering was left up to the discretion of the treating provider. Patients were excluded if they had a concomitant non-LRTI bacterial infection, expired within 3 days following sputum collection, had a tracheostomy present at the time of sputum collection, or had a contaminated/rejected specimen. Only first non-contaminated/rejected specimens per admission were included in the analysis. All samples were fresh clinical samples collected during routine clinical care and processed in accordance with the panel’s instructions for use (8). This study was approved by the Washington University in St. Louis Institutional Review Board with a waiver of informed consent.

### BFPP

Sputum specimens were analyzed on the BioFire FilmArray 2.0 using the BFPP. Testing was performed per the manufacturer’s instructions (8). Only semi-quantitative bacterial pathogens were analyzed, including the following: *Acinetobacter calcoaceticus-baumannii* complex, *Enterobacter cloacae* complex, *Escherichia coli*, *Haemophilus influenzae*, *Klebsiella aerogenes*, *Klebsiella oxytoca*, *Klebsiella pneumoniae* group, *Moraxella catarrhalis*, *Proteus* species*, Pseudomonas aeruginosa*, *Serratia marcescens*, *Staphylococcus aureus*, *Streptococcus agalactiae*, *Streptococcus pneumoniae*, and *Streptococcus pyogenes*. The following resistance determinants were reported qualitatively and were also analyzed: *bla*_CTX-M_, *bla*_IMP_, *bla*_KPC_, *bla*_NDM_, *bla*_OXA-48-like_, *bla*_VIM_, and *mecA/C* and MREJ.

### Sputum specimens

Sputum specimens with over 10 epithelial cells per low-power field on conventional Gram stain were considered contaminated with excessive oral flora and were rejected for BFPP and culture. Acceptable sputum specimens were tested by BFPP, quadrant struck on Trypticase soy agar with 5% sheep blood (BAP), chocolate agar (CHOC), and MacConkey agar (MAC) and were incubated at 35°C in 5% CO_2_. All plates were first examined for growth at 24 h of incubation. MAC plates without growth at 18–24 h were discarded; BAP and CHOC plates without growth at 48 h were discarded. Qualitative bacterial growth was reported as: “rare,” 10 colonies or fewer in the first quadrant; “few,” greater than 10 colonies in the first quadrant; “moderate,” greater than 10 colonies and growth into the second quadrant; “abundant,” heavy growth of colonies in the second quadrant, leading into growth in the third or fourth quadrant. Potentially colonizing bacteria, including *H. influenzae* and *S. pneumoniae*, were reported only if present in moderate or abundant quantities or if predominant. Cultures were reported as “upper respiratory flora” if only normal upper respiratory bacteria were isolated without significant pathogens.

### SOC culture

Isolated bacterial colonies were identified with a combination of biochemical testing and matrix-assisted laser desorption/ionization-time of flight mass spectrometry using the Bruker Biotyper. Susceptibility testing was performed using disk diffusion and gradient diffusion methods in accordance with Clinical and Laboratory Standards Institute (CLSI) standards, with results interpreted by CLSI M100 interpretive criteria (32–34th editions) (14–16). *S. aureus* isolates were classified as methicillin-resistant or methicillin-susceptible based on the Alere PBP2a SA culture colony test (Abbott Laboratory, Chicago, IL, USA) and disk diffusion testing of cefoxitin. The Cepheid (Sunnyvale, CA, USA) Xpert Carba-R assay was used as part of SOC testing from isolated colonies of *Enterobacterales*, which tested intermediate or resistant to any carbapenem tested for the detection of *bla*_IMP_, *bla*_KPC_, *bla*_NDM_, *bla*_OXA-48-like_, or *bla*_VIM_.

### Study definitions

For the purpose of exclusion, concomitant non-LRTI bacterial infection was defined as having one of the following within the 14 days preceding through 3 days following BFPP collection: (i) clinical suspicion of non-LRTI requiring antibiotics based on chart review and (ii) bacterial pathogen identification from another specimen source, with the exception of single blood cultures growing coagulase-negative *Staphylococcus* species and *Clostridioides difficile* infection. Patients were considered to have community-acquired LRTI if they were admitted for <48 h prior to sputum collection and hospital-acquired LRTI if they were admitted for ≥48 h prior to sputum collection. Active COVID-19 infection was defined as having a positive COVID-19 PCR within 10 days prior to or 4 days after sputum collection.

### Data and statistical analysis

Concordance between the BFPP to SOC culture results was calculated overall, by specimen type (induced or expectorated sputum), by antibiotic exposure, by semi-quantitative level, and for each bacterial target. Performance was calculated via standard methods and included positive percent agreement (PPA), negative percent agreement (NPA), positive predictive value (PPV), and negative predictive value (NPV). PPA was calculated using the number of concordant positive results divided by the total number of positive SOC culture results, while NPA was calculated using the number of concordant negative results divided by the total number of negative SOC culture results. PPA and NPA were calculated under the assumption that SOC culture results were the reference standard. PPV was calculated using the number of concordant positive results divided by the total number of BFPP positive results, while NPV was calculated using the number of concordant negative results divided by the total number of BFPP negative results. PPV and NPV were calculated under the assumption that the BFPP results were the reference standard. Baseline and antibiotic characteristics and outcomes were analyzed using descriptive statistics with mean and standard deviation or median and interquartile range (IQR) for continuous variables, as appropriate based on normality, and percentages for ordinal variables. Statistical analyses were performed using R version 4.4.1. Proportions were compared with Pearson’s chi-square or Fisher’s exact, as appropriate. Confidence intervals were calculated with the Pearson-Klopper method. A two-sided *P* value of <0.05 was considered statistically significant for all tests.

## RESULTS

### Patient population

**Fig 1:**
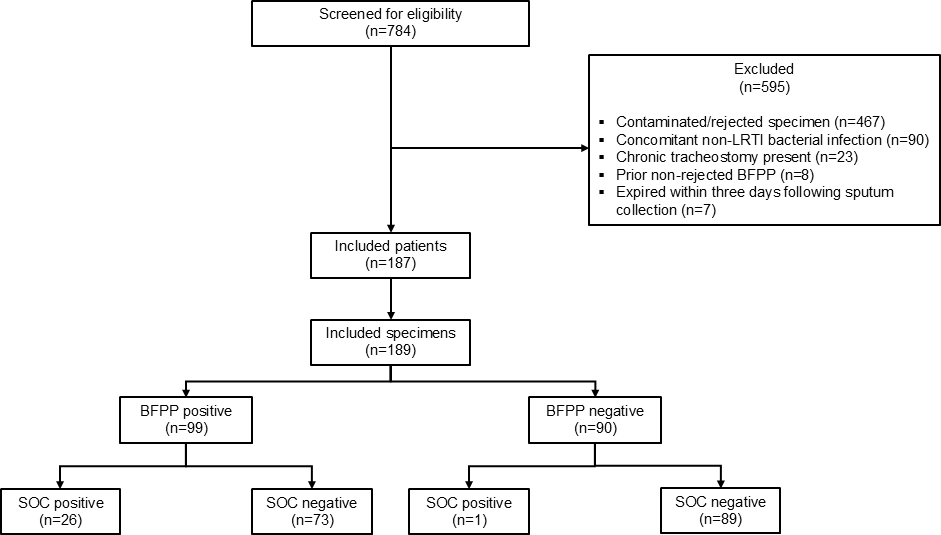
Study design. A total of 189 specimens from 187 adult patients between September 1, 2022, and August 31, 2024 were included.

A total of 784 sputum specimens were screened for eligibility, with 189 specimens from 187 unique patients meeting criteria for inclusion into the study and analysis (Fig. 1). The mean age of included patients was 60 years. Most patients were male (58.6%) and White (63%). The median Charlson comorbidity index was 3 (IQR 2–6), 28% of patients had chronic obstructive pulmonary disease, 31.7% diabetes, 16.7% hematologic malignancy, and 11.1% neutropenia at the time of sputum collection. Few patients had a history of a multidrug-resistant organism within 6 months prior to sputum collection, with the most common being *P. aeruginosa* (5.3%). Fifty percent of patients had a white blood cell count >10,000 cells/mcL and 16% of patients had a temperature ≥38°C within 24 h of sputum collection. Over half of patients required nasal cannula at the time of sputum collection, and the majority of LRTIs were classified as community-acquired (66.1%) as compared to hospital-acquired (33.9%). Most specimens were expectorated sputum (75.5%) and 24.5% were induced sputum. One hundred forty-four specimens (76.2%) were collected after antibiotic exposure with a median number of antibiotics of 1 (IQR 1–2) and a median duration of antibiotic exposure of 21.7 (IQR 8.7–38.2) hours prior to sputum collection. The most commonly administered antibiotics prior to sputum collection were cefepime (36%), vancomycin (31.2%), and azithromycin (25.5%) (Table 1).

**TABLE 1:** Patient and antibiotic characteristics^*b*^^,^^*e*^

Patient characteristic	*N* = 189^*a*^
Age, years, mean (SD)	60.1 (14.6)
Male, *n* (%)	109 (58.6)
Race, *n* (%)	
White	119 (63)
Black	61 (32.3)
Other	6 (3.2)
Unable to answer/declined	3 (1.6)
Season of testing, *n* (%)	
Winter (December–February)	44 (23.4)
Spring (March–May)	67 (35.6)
Summer (June–August)	43 (22.9)
Fall (September–November)	35 (18.6)
Charlson comorbidity index, median (IQR)	3 (2–6)
Select comorbidities, *n* (%)	
Asthma	19 (10)
Chronic obstructive pulmonary disease	53 (28)
Diabetes	60 (31.7)
Immunocompromising condition, *n* (%)	
Solid organ transplant	6 (3.2)
Hematologic malignancy	31 (16.4)
Hematopoietic cell transplant	11 (5.8)
People with HIV	8 (4.2)
Neutropenia (ANC < 1,000 cells/mcL)	21 (11.1)
History of MDR infection/colonization within 6 months of sputum collection, *n* (%)	
Extended-spectrum beta-lactamase Enterobacterales	1 (0.5)
Carbapenem-resistant Enterobacterales	0 (0)
Carbapenem-resistant *Acinetobacter calcoaceticus-baumannii* complex	0 (0)
*Pseudomonas aeruginosa*	10 (5.3)
Methicillin-resistant *Staphylococcus aureus*	2 (1.1)
Infectious signs/symptoms within 24 h of sputum collection, *n*/*N* (%)	
WBC > 10,000 cells/mcL	90/180 (50)
WBC < 4,000 cells/mcL	33/180 (18.3)
Temperature ≥ 38°C	30/188 (16)
Temperature ≤ 36°C	7/188 (3.7)
Location at sputum collection, *n* (%)	
Emergency department	8 (4.2)
Floor	181 (95.8)
Hospital LOS prior to sputum order, days, median (IQR)	1.1 (0.3–3.0)
Hospital LOS prior to sputum collection, days, median (IQR)	1.5 (0.6–3.5)
Respiratory support at time of sputum collection, *n* (%)	
None	95 (50.3)
Nasal cannula	94 (49.7)
Specimen type, *n* (%)	
Expectorated sputum	142 (75.5)
Induced sputum	47 (24.5)
Suspected infection type, *n* (%)	
Community-acquired LRTI	125 (66.1)
Hospital-acquired LRTI	64 (33.9)
Active COVID-19 infection, *n* (%)	8 (4.2)
Infectious diseases consult during hospitalization, *n* (%)	51 (27)

Antibiotic characteristic	*N* = 189
Antibiotic exposure prior to sputum collection, *n* (%)	144 (76.2)
Duration of antibiotic exposure prior to sputum collection, hours, median (IQR)	21.7 (8.7–38.2)
Antibiotics at sputum collection, *n* (%)^*c*^	
Ampicillin/sulbactam	6 (3.2)
Azithromycin	48 (25.4)
Cefepime	68 (36)
Ceftriaxone	35 (18.5)
Levofloxacin	5 (2.6)
Linezolid	9 (4.8)
Meropenem	13 (6.9)
Piperacillin/tazobactam	10 (5.3)
Vancomycin	59 (31.2)
Other^*d*^	11 (5.8)
Number of antibiotics at sputum collection, median (IQR)	1 (1–2)
LOS following BFPP result, days, median (IQR)	4.1 (2.1–7.4)

^*a*^ A total of 189 sputum specimens from 187 patients; 2 patients were included two times due to meeting criteria during two distinct admissions.

^*b*^ Immunocompromising conditions were not considered mutually exclusive.

^*c*^ Percentages add up to greater than 100 due to patients being on multiple antibiotics concurrently.

^*d*^ Other antibiotics: amoxicillin/clavulanate (*n* = 2), aztreonam (*n* = 1), cefiderocol (*n* = 1), ceftriaxone (*n* = 1), doxycycline (*n* = 3), minocycline (*n* = 2), and moxifloxacin (*n* = 1).

^*e*^ ANC, absolute neutrophil count; BFPP, BioFire FilmArray Pneumonia Panel; HIV, human immunodeficiency virus; IQR, interquartile range; LOS, length of stay; LRTI, lower respiratory tract infection; MDR, multidrug-resistant; SD, standard deviation.

### Bacterial target detection

A total of 141 bacterial targets were detected by the BFPP from 189 sputum specimens. At least one bacterial target was detected by the BFPP in 52.4% of specimens overall (*n* = 99/189). The most frequent bacterial targets detected were *H. influenzae* (20.1%), *P. aeruginosa* (13.2%), *S. aureus* (11.6%), and *S. pneumoniae* (11.6%). Co-detections occurred in 31.3% of specimens, with 23.2% of positive specimens detecting two bacterial targets, 6.1% of positive specimens detecting three bacterial targets, and one positive specimen each detecting four and five targets. The most frequent co-detections were *S. aureus* (15/31 specimens), *H. influenzae* (14/31 specimens), *S. pneumoniae* (11/31 specimens), and *M. catarrhalis* (8/31 specimens) (Table 2). Rates of bacterial targets were similar between community-acquired and hospital-acquired LRTI patients, except for more *S. pneumoniae* detections in community-acquired LRTI patients (15.2% vs 4.7%, *P* = 0.03) and more *E. cloacae* complex detections in HAP patients (6.3% vs 0%, *P* = 0.01). BFPP did not detect *A. calcoaceticus-baumannii* complex, *K. aerogenes*, or *K. oxytoca* in any specimens (Table 3).

**TABLE 2:** Number of BFPP bacterial detections per specimen^*a*^

BFPP result	All sputum		Expectorated sputum		Induced sputum	
	(*N* = 189)		(*n* = 142)		(*n* = 47)	
	Number detected	% of Total (% of positive)	Number detected	% of Total (% of positive)	Number detected	% of Total (% of positive)
Total positive specimens	99	52.4	79	55.6	20	42.6
One detection	68	36 (68.7)	53	37.3 (67.1)	15	31.9 (75)
Two detections^*b*^	23	12.2 (23.2)	21	14.8 (26.6)	2	4.3 (10)
Three detections^*c*^	6	3.2 (6.1)	4	2.8 (5.1)	2	4.3 (10)
Four detections^*d*^	1	0.5 (1)	0	0	1	2.1 (5)
Five detections^*e*^	1	0.5 (1)	1	0.7 (1.2)	0	0

^*a*^ BFPP, BioFire FilmArray Pneumonia Panel.

^*b*^ Co-detections include: *H. influenzae + S. pneumoniae* (*n* = 5), *H. influenzae + S. aureus* (*n* = 3), *P. aeruginosa + S. aureus* (*n* = 3), *K. pneumoniae* group + *S. aureus* (*n* = 1), *Proteus* species + *S. aureus* (*n* = 1), *S. agalactiae + S. aureus* (*n* = 1),* S. pyogenes + S. aureus* (*n* = 1), *H. influenzae + E. coli* (*n* = 1), *H. influenzae + M. catarrhalis* (*n* = 1), *H. influenzae + P. aeruginosa* (*n* = 1), *M. catarrhalis + P. aeruginosa* (*n* = 1), *M. catarrhalis + S. pneumoniae* (*n* = 1), *S. pneumoniae + S. marcescens* (*n* = 1), *P. aeruginosa + E. cloacae* complex (*n* = 1), *P. aeruginosa + E. coli* (*n* = 1).

^*c*^ Co-detections include: *E. cloacae* complex + *S. agalactiae + S. aureus* (*n* = 1), *M. catarrhalis + S. aureus + S. pneumoniae* (*n* = 1), *H. influenzae + S. agalactiae + S. aureus* (*n* = 1), *M. catarrhalis + S. marcescens + S. pneumoniae* (*n* = 1), *E. coli + K. pneumoniae* group + *S. aureus* (*n* = 1), *M. catarrhalis + P. aeruginosa + S. marcescens* (*n* = 1).

^*d*^ Co-detections include: *H. influenzae + M. catarrhalis + P. aeruginosa + S. pneumoniae* (*n* = 1).

^*e*^ Co-detections include: *H. influenzae + M. catarrhalis + P. aeruginosa + S. aureus + S. pneumoniae* (*n* = 1).

**TABLE 3:** Distribution of BFPP bacterial target detections by suspected LRTI type^*a*^^,^^*b*^

Bacterial target, *n* (%)	Community-acquired (*n* = 125)	Hospital-acquired (*n* = 64)	*P* value	Total (*N* = 189)
*Staphylococcus aureus*	13 (10.4%)	9 (14.1%)	0.61	22 (11.6%)
MSSA	7 (5.6%)	5 (7.8%)	0.75	12 (6.3%)
MRSA	6 (4.8%)	4 (6.3%)	0.74	10 (5.3%)
*Streptococcus agalactiae*	3 (2.4%)	2 (3.1%)	>0.99	5 (2.6%)
*Streptococcus pneumoniae*	19 (15.2%)	3 (4.7%)	**0.03**	22 (11.6%)
*Streptococcus pyogenes*	1 (0.8%)	0	>0.99	1 (0.5%)
*Acinetobacter calcoaceticus-baumannii* complex	0	0	–^*c*^	0
*Enterobacter cloacae* complex	0	4 (6.3%)	**0.01**	4 (2.1%)
*Escherichia coli*	2 (1.6%)	1 (1.6%)	>0.99	3 (1.6%)
*Haemophilus influenzae*	25 (20%)	13 (20.3%)	>0.99	38 (20.1)
*Klebsiella aerogenes*	0	0	–	0
*Klebsiella oxytoca*	0	0	–	0
*Klebsiella pneumoniae* group	1 (0.8%)	2 (3.1%)	0.26	3 (1.6%)
*Moraxella catarrhalis*	10 (8%)	1 (1.6%)	0.10	11 (5.8%)
*Proteus* species	1 (0.8%)	0	>0.99	1 (0.5%)
*Pseudomonas aeruginosa*	16 (12.8%)	9 (14.1%)	0.99	25 (13.2%)
*Serratia marcescens*	4 (3.2%)	1 (1.6%)	0.66	5 (2.6%)

^*a*^ BFPP, BioFire FilmArray Pneumonia Panel; LRTI, lower respiratory tract infection.

^*b*^ Bolded values are considered statistically significant.

^*c*^ Dashes indicate that no statistical comparisons were made since there were no patients in either group with that organism.

### Test performance

Characteristics and concordance between BFPP and SOC culture are presented in Table 4. Twenty-six (13.8%) specimens had a bacterial target detected on BFPP that was concordant with growth on SOC culture, 73 (38.6%) specimens had a bacterial target detected on BFPP with no concordant growth on SOC culture, 89 (47.1%) specimens did not have a bacterial target detected on BFPP or growth on SOC culture, and 1 specimen had bacterial growth on SOC culture that was not detected on BFPP. This resulted in an overall PPA of 96.3%, NPA of 54.9%, PPV of 26.3%, and NPV of 98.9%. Test performance was similar when analyzed by specimen type and suspected infection type. When analyzed by antibiotic exposure, patients with greater than 24 h of exposure prior to sputum collection had a lower PPV compared to patients with less than 24 h or no exposure prior (13.6% vs 29.6% vs 30.4%).

**TABLE 4:** Characteristics and concordance of BFPP results with SOC culture results^*d*^

Characteristic	BFPP+SOC+	BFPP+SOC−	BFPP−SOC−	BFPP−SOC+	PPV	NPV	PPA	NPA
Overall	26	73	89	1	26.3% (17.9–36.1)	98.9% (94.0–100)	96.3% (81.0–99.9)	54.9% (46.9–62.8)
Overall by sputum type								
Induced	6	14	27	0	30% (11.9–54.3)	100% (87.2–100)	100% (54.1–100)	65.9% (49.4–79.9)
Expectorated	20	59	62	1	25.3% (16.2–36.4)	98.4% (91.5–100)	95.2% (76.2–99.9)	51.2% (42.0–60.4)
Overall by suspected infection type								
Community-acquired	17	50	57	1	25.4% (15.5–37.5)	98.3% (90.8–100)	94.4% (72.7–99.9)	53.3% (43.4–63.0)
Hospital-acquired	9	23	32	0	28.1% (13.7–46.7)	100% (89.1–100)	100% (66.4–100)	58.2% (44.1–71.3)
Overall by antibiotic exposure								
>24 h	3	19	39	0	13.6% (2.9–34.9)	100% (91.0–100)	100% (29.2–100)	67.2% (53.7–79.0)
≤24 h	16	38	28	1	29.6% (18.0–43.6)	96.6% (82.2–99.9)	94.1% (71.3–99.9)	42.4% (30.3–55.2)
No antibiotic exposure	7	16	22	0	30.4% (13.2–52.9)	100% (84.6–100)	100% (59.0–100)	57.9% (40.8–73.7)
Semi-quantitative BFPP^*a*^								
10^4^	4	30	-	-	11.8% (3.3–27.5)	-	-	-
10^5^	1	34	-	-	2.9% (0.1–14.9)	-	-	-
10^6^	5	22	-	-	18.5% (6.3–38.1)	-	-	-
10^7^	26	19	-	-	57.8% (42.2–72.3)	-	-	-
Bacterial target^*a*^								
*Staphylococcus aureus*	5	17	167	1	22.7% (7.8–45.4)	99.4% (96.7–100)	83.3% (35.9–99.6)	90.8% (85.6–94.5)
MSSA	1	11	177	1	8.3% (0.2–38.5)	99.4% (96.9–100)	50% (1.3–98.7)	94.1% (89.8–97.0)
MRSA	4	6	179	0	40% (12.2–73.8)	100% (98.0–100)	100% (39.8–100)	96.8% (93.1–98.8)
*Streptococcus agalactiae*	1	4	184	0	20% (0.5–71.6)	100% (98.0–100)	100% (2.5–100)	97.9% (94.6–99.4)
*Streptococcus pneumoniae*	4	18	167	0	18.2% (5.2–40.3)	100% (97.8–100)	100% (39.8–100)	90.3% (85.1–94.1)
*Streptococcus pyogenes*	1	0	188	0	100% (2.5–100)	100% (98.0–100)	100% (2.5–100)	100% (98.0–100)
*Acinetobacter calcoaceticus-baumannii* complex	0	0	189	0	-^*b*^	100% (98.0–100)	--^*c*^	100% (98.9–100)
*Enterobacter cloacae* complex	1	3	185	0	25% (0.6–80.6)	100% (98.0–100)	100% (2.5–100)	98.4% (95.4–99.7)
*Escherichia coli*	0	3	185	0	0% (0–70.8)	100% (98.0–100)	--^*c*^	98.4% (95.4–99.7)
*Haemophilus influenzae*	6	33	150	0	15.4% (5.9–30.5)	100% (97.6–100)	100% (54.1–100)	82% (75.6–87.2)
*Klebsiella aerogenes*	0	0	189	0	-^*b*^	100% (98.0–100)	--^*c*^	100% (98.0–100)
*Klebsiella oxytoca*	0	0	189	0	-^*b*^	100% (98.0–100)	--^*c*^	100% (98.0–100)
*Klebsiella pneumoniae* group	1	2	186	0	33.3% (0.8–90.6)	100% (98.0–100)	100% (2.5–100)	98.9% (96.2–99.9)
*Moraxella catarrhalis*	2	9	178	0	18.2% (2.3–51.8)	100% (97.9–100)	100% (15.8–100)	95.2% (91.1–97.8)
*Proteus* species	1	0	188	0	100% (2.5–100)	100% (98.0–100)	100% (2.5–100)	100% (98.0–100)
*Pseudomonas aeruginosa*	12	13	164	0	48% (27.8–68.7)	100% (97.8–100)	100% (73.5–100)	92.7% (87.8–96.0)
*Serratia marcescens*	2	3	184	0	40% (5.3–85.3)	100% (98.0–100)	100% (15.8–100)	98.4% (95.4–99.7)

^*a*^ Includes all detections from BioFire FilmArray Pneumonia Panel.

^*b*^ Cannot be determined as the organism was never detected on the BioFire FilmArray Pneumonia Panel and never grew in standard of care culture.

^*c*^ Cannot be determined as the organism never grew in standard of care culture.

^*d*^ BFPP, BioFire FilmArray Pneumonia Panel; MRSA, methicillin-resistant *Staphylococcus aureus*; MSSA, methicillin-susceptible *Staphylococcus aureus*; NPA, negative percent agreement; NPV, negative predictive value; PPA, positive percent agreement; PPV, positive predictive value; SOC, standard of care.

When analyzed by specific bacterial targets, PPV ranged from 0% to 100%, NPV ranged from 99.4% to 100%, PPA ranged from 50% to 100%, and NPA ranged from 82% to 100%. The lowest concordance between BFPP and SOC culture was observed for *H. influenzae* (15.4%), *M. catarrhalis* (18.2%), *S. pneumoniae* (19%), and *S. aureus* (22.7%). BFPP showed a high NPA, with all bacterial targets having an NPA greater than 90%, except for *H. influenzae* (82%). A single on-panel bacterial target (methicillin-susceptible *S. aureus* [MSSA]) was reported as rare growth by SOC culture, but not detected by BFPP. Upon review, in these quantities, the MSSA should have been included in upper respiratory flora per routine lab protocol. Four off-panel bacteria and one off-panel fungus were detected by SOC culture, including *Corynebacterium accolens* (*n* = 1)*, Streptococcus dysgalactiae* (*n* = 1)*, Streptococcus canis* (*n* = 1)*, Stenotrophomonas maltophilia* (*n* = 2), and *Rhizomucor* (*n* = 1).

Concordance between BFPP and SOC culture was highest among specimens with a semi-quantitative BFPP titer of ≥10^7^ copies/mL (57.8%). Most bacterial detections with semi-quantitative BFPP titers of 10^6^, 10^5^, and 10^4^ copies/mL did not have growth on SOC culture (Table 3). The distribution of BFPP results compared to qualitative SOC culture results is in Table 5.

**TABLE 5:** Distribution of BFPP results compared to qualitative SOC culture result^*a*^

BFPP result	Upper respiratory flora	Number of specimens with qualitative SOC culture result			
		Rare	Few	Moderate	Abundant
Not detected	88	1	1	0	1
10^4^ copies/mL	28	3	1	2	0
10^5^ copies/mL	33	0	2	0	0
10^6^ copies/mL	22	1	1	3	0
≥10^7^ copies/mL	19	0	2	12	12

^*a*^ BFPP, BioFire FilmArray Pneumonia Panel; SOC, standard of care.

### Detection of antimicrobial resistance

Ten specimens were positive for *mecA/C* and MREJ with concordant growth of MRSA on SOC culture in four specimens; the remaining six specimens did not have MRSA growth on SOC culture. One specimen was positive for *bla*_CTX-M_ with no concordant growth on SOC culture; *bla*_IMP_, *bla*_KPC_, *bla*_NDM_, *bla*_OXA-48-like_, and *bla*_VIM_ were not detected in any specimens.

## DISCUSSION

Previous published literature on real world BFPP performance often focuses on deeper respiratory specimens, combines all specimen types or combines sputum and ETA specimens for analyses, making the analytical performance of the BFPP on solely sputum specimens less clear (6, 7, 17–22). This study evaluated the analytical performance of the BFPP as compared to SOC culture from 189 sputum specimens from non-ICU patients with suspected LRTI, which, to our knowledge, is the largest analysis of exclusively expectorated or induced sputum specimens in this patient population published to date.

In our study, a bacterial target was identified by BFPP in 52.4% of specimens, with bacterial co-detections occurring in 31.3% of specimens. Our data show the BFPP had an overall high PPA with SOC culture of 96.3% but an overall low PPV of 26.3% and NPA of 54.9% due to the BFPP detecting additional bacteria not recovered on SOC culture. These additional detections were mostly *H. influenzae*, *M. catarrhalis*, *S. pneumoniae*, and *S. aureus*, which colonize the respiratory tract and may be indicative of normal flora and not necessarily the causative pathogen of LRTI. Higher rates of retrieval of these organisms from BFPP compared to SOC culture are consistent with previously published studies, including a study done across all specimen types at our institution (17, 20, 22–25). Overall test performance was similar when analyzed by specimen and suspected infection type, though some subgroup analyses had small sample sizes.

A recent similar evaluation including both ICU and non-ICU patients was completed by Falsey et al., with 96% of included specimens being expectorated sputum and 4% being ETA specimens. Both moderate quality (>25 polymorphonuclear [PMN] cells per high-power field [hpf] and >10 epithelial cells/hpf) and good quality (>25 PMN/hpf and <10 epithelial cells/hpf) sputum specimens were included. Two hundred three good quality sputum specimens were included, with an overall concordance between the BFPP and SOC culture results of 37%, which is higher than the overall concordance of 26.3% seen in the present study. When analyzed by antibiotic use, 44% of patients with no antibiotic use had results concordant with SOC compared to 32% of patients with antibiotic use, with a median time from administration to specimen collection of 10 h (26). This lower concordance in patients with antibiotic exposure was also noted in our study for those patients who were exposed to greater than 24 h of antibiotics (13.6%) compared to those who were not (30.4%). Antibiotic pre-exposure leading to discordances is corroborated by a recent *in vitro* study of BFPP and SOC with standardized antibiotic exposures and bacteria inocula with results showing 51% of gram-negative isolates when exposed to antibiotics were reported as no growth or no significant growth by SOC but still detected by BFPP (27). Finally, both the study of Falsey et al. as well as ours found a high NPV, highlighting the good performance of BFPP as a rule-out test.

Our microbiology laboratory qualitatively reports SOC culture growth for sputum specimens, which limits a direct comparison between copies/mL and colony-forming units/mL; however, the distribution of BFPP results compared to qualitative SOC culture results was evaluated. Bacterial targets that were detected at 10^6^ or ≥10^7^ copies/mL were less likely to be reported as “upper respiratory flora” than those that were detected at 10^4^ or 10^5^ copies/mL, which may be due to the BFPP detecting the DNA of dead bacteria or extracellular DNA for those lower level threshold detections, our microbiology laboratory’s reporting procedures for sputum specimens, fastidious bacteria, or the effect of antibiotic exposure prior to sputum collection. The correlation between semi-quantitative BFPP thresholds and clinical relevance deserves further investigation.

Several limitations to our study exist. This was a retrospective, single-center study at a large, 1400-bed academic medical center focused on the bacterial performance of the BFPP only; the epidemiology of bacterial targets seen at our institution, including the low number of antimicrobial resistance target detections, may limit external validity. Moreover, given the setting as well as the population observed in our cohort (e.g., 27% infectious diseases consults), our population may be sicker and more immunocompromised than some other settings using BFPP. Due to the lack of reference methods for viral or atypical organisms, our performance comparisons did not include those data. Future studies among non-ICU patients should examine this. Similarly, we only compared to SOC without reference method comparison or discrepancy analysis, thus making our performance data more real world, which may differ from regulatory performance studies. The high rate of excluded sputum specimens, mainly due to contaminated/rejected specimens, led to small sample sizes for some subgroups, specifically induced sputum specimens and some of the individual bacterial targets, which makes interpretation of concordance between BFPP and SOC culture less clear. Additional inclusion criteria of clinical symptoms and/or radiographic findings of LRTI may have improved selection of patients with true LRTI, leading to a decrease in the number of BFPP positive SOC culture negative results. We also excluded patients who expired within 3 days to ensure a homogenous low acuity population given previous BFPP research has focused on severe LRTI. While this may have limited our conclusion, this exclusion (*n* = 7) reflected only 1% of eligible patients evaluated and thus is unlikely to have impacted our analysis in any substantive manner. Our specimen population was limited to high-quality sputum specimens, which should have helped to improve specimen selection, though challenges are still seen. However, these results reflect real-world performance based on routine clinical ordering, and thus future work with this highly sensitive test should focus on diagnostic stewardship with biomarkers or algorithms akin to *C. difficile* PCR testing (28).

In summary, usage of the BFPP in sputum specimens from non-ventilated, floor-level patients led to an overall high PPA and NPV but a low NPA and PPV. Given this, the BFPP could be beneficial when attempting to rule out a bacterial LRTI, but the benefit is less clear when there is a positive BFPP result, especially when a common airway colonizer is detected at low semi-quantitative thresholds. The diagnosis of LRTI is not solely based on microbiologic detection, but also on clinical symptoms, which should be relied on when interpreting BFPP results in relation to SOC culture results.

## Data Availability

All relevant data are contained within the manuscript.
